# Adapting Measures of Anxiety and Mood Disorders for Use with Autistic Adults: A Systematic Review

**DOI:** 10.1007/s40474-026-00348-3

**Published:** 2026-03-16

**Authors:** Ellen Wilkinson, Alexis M. Brewe, Richard P. Hastings, Andrew Jahoda, Susan W. White, Vanessa H. Bal

**Affiliations:** 1 Department of Psychology, Rutgers University, New Brunswick, USA; 2 TEACCH Autism Program, University of North Carolina at Chapel Hill, Chapel Hill, United States; 3 School of Social Policy and Society, University of Birmingham, Birmingham, United Kingdom; 4 Mental Health and Wellbeing, School of Health and Wellbeing, University of Glasgow, Glasgow, United Kingdom; 5 Center for Youth Development and Intervention, University of Alabama, Tuscaloosa, United States; 6 Graduate School of Applied and Professional Psychology, Rutgers University, Piscataway, United States

## Abstract

**Purpose of Review:**

The aim of this systematic review is to investigate the adaptations made to existing anxiety and mood disorder measures used with autistic adults. It addresses the types of modifications made to such measures, the processes behind them, and the involvement of autistic individuals.

**Recent Findings:**

Following PRISMA guidelines, the review searched four major databases for studies published in English since 1994 that used mood or anxiety measures with autistic adults. Out of 14,583 identified studies, 32 met the inclusion criteria for reporting adaptations. Data extraction used modified FRAME and GRIPP2 frameworks to capture the nature and justification of adaptations and the role of stakeholder involvement.

**Summary:**

The review found that only 8% of studies using mood or anxiety measures with autistic adults reported any adaptations, most of which involved using tools developed for children or converting self-report measures to proxy-report without clear justification. Very few studies included psychometric validation of the adapted tools, and only one study explicitly involved autistic adults in the adaptation process. The findings highlight a critical need for transparency in reporting adaptations, involvement of autistic individuals in tool development, and validation of adapted instruments to ensure accurate mental health assessment in autistic populations.

**Supplementary Information:**

The online version contains supplementary material available at 10.1007/s40474-026-00348-3.

## Introduction

Anxiety and mood disorders have consistently been found to occur more frequently among autistic adults than in the general population [1], highlighting the need to be able to identify and measure such disorders in autistic people. New measures specifically for use with autistic adults are being developed (e.g., Autistic Depression Assessment Tool–Adult [2], . Nonetheless, existing instruments designed for use in the general population are commonly used to screen and/or diagnose these conditions in autistic adults [3, 4]. Despite some references to “adaptations” or “accommodations” of instruments when used with autistic people [4], the changes that are being implemented to make these instruments more suitable or valid for autistic adults have not been systematically described.

While accessibility (e.g., complex versus simple language) is one clear reason for adaptation, the rationale for adapting instruments extends beyond measurement accessibility alone. Some adaptations address purely measurement-related barriers (e.g., wording, response formats), whereas other issues reflect deeper questions about whether the mechanisms, experiences, or manifestations of anxiety and mood disorders in autism differ from those observed in the general population. Clarifying these distinctions is essential for interpreting why specific adaptations are needed.

### Rationale for Instrument Adaptations for Autistic Adults

There are many reasons why adaptation or development of new instruments for autistic adults may be warranted. First, autistic individuals may experience emotions differently from the general population, with studies finding many autistic people have difficulty identifying their own emotions [5, 6]. It is thought that approximately half of autistic people experience alexithymia [7]. Therefore, autistic adults are more likely to have difficulty answering questions about their emotions and behaviors as they specifically relate to mood and anxiety. A study examining the relationships between alexithymia, autism-related socio-communication difficulties, and anxiety/depression symptoms found that difficulty in identifying feelings was strongly positively associated with anxiety symptoms and self-reported social difficulties [8].

Secondly, the manifestation of anxiety and depression in autism may be different from symptoms used to characterize these conditions in the general population. Compared to others, when experiencing depression, autistic people tend to report an increase in sensory sensitivity, agitation and decreased absorption by a special interest [9]. Complicating this, increased obsessive thoughts and behaviors, including special interests, have also been reported to increase with depression [10]. Several studies have demonstrated that anxiety symptoms in autistic people are closely linked to core autism characteristics. For instance, autistic people have described their anxiety as a fear of consequences when they don’t understand a social interaction, or unease caused by not understanding others’ intentions [11, 12]. Anxiety is also sometimes related to restricted and repetitive behaviors. Some people indicate that their anxiety is driven by a change in plans or not being able to engage in certain actions (i.e. stim; [11, 13]). These patterns of symptoms are not described in the standard DSM-5 criteria for anxiety and depression [14] and are not included in screening or diagnostic tools designed for use in the general population. These differences in manifestation reflect conceptual issues, namely whether anxiety and mood disorders in autism arise from the same, partially overlapping, or distinct mechanistic pathways as in non-autistic populations. Thus, some adaptations may aim to reflect autistic experiences of mental health more accurately, rather than simply improving accessibility of existing items.

Beyond differences in manifestation of co-occurring mood and anxiety conditions, there is also evidence to suggest that there is a high degree of overlap between features of autism and symptoms of depression, anxiety, and bipolar disorder. For example, intolerance of uncertainty [15] is a feature of anxiety that is also commonly observed in autistic people. Impairments in adaptive behavior, such as personal hygiene, and differences in sleep and eating, can be found in both depression and autism [10]. Difficulty expressing emotional states, social withdrawal, and restlessness are commonly observed in autism but are also associated with bipolar disorder [16]. Thus, items intended to capture symptoms of mood or anxiety disorders may be frequently endorsed due to overlap with autistic features and could artificially inflate scores and reduce instrument specificity.

Social-communication differences in autism may also warrant consideration in the adaptation of screening and diagnostic tools for depression. Autistic people tend to process language literally [17], necessitating careful consideration of how instructions and items are worded. For example, many response options employ a Likert scale, in which ratings are made in a linear fashion with anchors such as “Never” to “Often”. If an autistic person experienced the emotion in question once in the last 6 months, they may struggle to decide whether that one instance should be classified as never or some intermediary value (e.g., sometimes) due to a lack of specificity in instructions [18]. These represent measurement-focused adaptations, aimed at improving clarity, precision, and accessibility regardless of underlying symptom mechanisms.

Finally, it is important to acknowledge the wide range of cognitive abilities represented within the autistic population. Autistic people with intellectual disabilities historically have not been asked to complete self-report instruments due to assumptions that these individuals cannot respond and the lack of instruments with accessible language [19, 20]. A common adaptation to address this is to administer a self-report survey to another informant like a parent, asking the parent to complete it from the perspective of their autistic adult (i.e., to make their best estimate of how this person would respond; [21]). Alternatively, a measure may be changed to specifically ask for the parent’s perspective on the person’s thoughts and behaviors [22, 23]. A third alternative is to keep administration as self-report and use the child version of an instrument, affording simpler wording or a focus on features that may be more aligned with symptoms of depression in people with intellectual disabilities. For example, irritability is considered a symptom of depression in children (DSM-5), which has also been characteristic of depression in those with intellectual disabilities [24] and minimally speaking adults with autism [25].

Taken together, there are many factors that suggest a potential need for measurement adaptations. Adaptations may focus on item content, ranging from consideration of the indicators of mood and anxiety conditions in autistic people to the elimination of non-relevant symptoms for this population. Cognitive interviewing to ensure interpretability of items and to identify potential problems in response options has been used in the development of other types of measures for autistic children and adults [26–28]. This methodology will be critical to inform adaptation and development of mood and anxiety screeners and diagnostic tools. Beyond content, adaptations may also require attention to format options, score distribution, and potential generation of “norms” and clinical cutoffs for autistic people. Importantly, adaptations may serve distinct purposes: increasing accessibility and appropriateness of existing scales, ensuring that items reflect autistic lived experience, or developing new measures that capture potentially diverse manifestations of mental health in autism. Making these distinctions explicit helps clarify the conceptual basis for instrument modification.

### The Current Study

While there is ample justification for adaptation of instruments, what adaptations are employed and their subsequent impact on psychometric properties of the instruments when used with autistic adults are not yet well-understood. Most existing mood and anxiety measures were not developed by or with people with lived experience of autism (i.e., autistic people). This is particularly problematic, as measurement tools developed solely based on behavioral observations by second parties (e.g., clinicians or caregivers) may fail to capture the unique internal experiences of the person, which could be critical to accurate diagnostic evaluation. It is therefore important to involve autistic adults in the adaptation of diagnostic and screening instruments. To create valid measures for this population, it is also important for the researchers making the adaptations to transparently document how autistic adults were involved. Such inclusion is essential to ensure the validity of newly developed or adapted instruments for assessing mood and anxiety disorders in autistic people. By better describing the features that autistic people identify as either relevant or not relevant to their experience, the theory behind these disorders becomes better informed, improving measurement.

Thus, the focus of this systematic review is to explore the adaptations made to instruments used to assess anxiety and mood disorders in studies of autistic adults. Although there exist systematic reviews of the availability and psychometric properties of anxiety and mood measures for autistic children or adults [4, 29, 30], we could find no existing reviews of *adaptations* to such measures. By systematically describing adaptations made to instruments, this review will yield information essential to contextualize the burgeoning literature on mental health in autism and inform future use, adaptation, and development of instruments to evaluate mood and anxiety disorders in autistic adults. To do this, this review aims to address three overarching questions:

- Question 1 – What adaptations have been made to existing measures of anxiety and mood for autistic adults?
- Question 2 – What has been the adaptation process?
- Question 3 - How were autistic adults involved?

## Methods

### Search Strategy

The review protocol was registered online with PROSPERO (CRD42023383648). The Preferred Reporting Items for Systematic Reviews and Meta-Analysis (PRISMA) guidelines were followed throughout the review process. In consultation with a library scientist, the following databases were searched for publications: PubMed, PsycInfo, MEDLINE (EBSCOHOST), MEDLINE (OVID). A search strategy was developed, with filters enabled to target articles published in the English language from January 1994, to identify articles meeting criteria described below. The original search was run on January 18th, 2023. An updated search employing the same search methods was run on July 22, 2024. Duplicates were removed using Covidence’s duplication identification software for both rounds of searches. Duplicates not identified by Covidence (e.g., a sample represented in both a published dissertation and a peer-reviewed journal) were removed by EW during the screening process.

## Inclusion and Exclusion Criteria

The study included articles that met the following inclusion criteria:

1. described a sample or subsample that was at least 70% diagnosed with autism spectrum disorder, Asperger’s syndrome, or pervasive developmental disorder-not otherwise specified.
2. had a mean age of at least 18 years.
3. published in 1994 or later, as that is when the DSM-IV published criteria comparable to the current day.
4. included a measure of anxiety and/or mood. Anxiety and mood disorders were operationalized as any anxiety or mood disorder via the DSM-5 (e.g., Generalized Anxiety Disorder, Major Depressive Disorder) and their broader conceptions (e.g., anxiety, depression).
5. Written in English, though the adapted measure did not need to be in English.

Studies were excluded if they met the following exclusion criteria:

1. did not include original empirical findings (e.g., opinion, conceptual pieces).
2. sample was defined by “autistic traits” (as opposed to autism diagnosis).
3. included only surveys measuring constructs related to anxiety and mood disorders but not a specific disorder (e.g., emotion regulation).

Of note, the 70% specifier for inclusion criterion 1 was selected to ensure that the results are meaningfully representative of autistic populations and not diluted by heterogeneous diagnostic groups. This quantitative threshold enhances the interpretability and external validity of the review’s conclusions while providing a transparent and reproducible standard for study selection. All identified studies had a sample entirely composed of autistic individuals or contained an independetly-described subsample of autistic individuals (e.g [31]). , . Prior to review of the identified studies, it was decided that self-diagnosed individuals would not be included in the diagnostic autistic groups. This specification was not relevant to any reviewed studies. Additionally, some individuals under 18 years of age are included due to inclusion criterion 2 above. This decision was made so as not to be overly exclusive and truly capture the measures being used with adults.

## Screening Procedure

Author EW screened the titles and abstracts of all records using the specified inclusion and exclusion criteria. Author AB reviewed 50% of the titles and abstracts. Discrepancies in screening were discussed, and a consensus was reached. The interrater agreement was acceptable (Cohen’s kappa=0.85). Full texts were then obtained and assessed for eligibility. Again, EW screened all full texts, and AB reviewed 50% of the articles. Discrepancies were discussed, and a consensus was reached. The interrater agreement was acceptable (Cohen’s kappa=0.65; [32, 33]). The relatively low agreement was primarily driven by the first few dozen articles, after which EW and AB met and fine-tuned the review process through in-depth discussion and live review of discrepancies. The agreement after this was higher, though not able to be calculated due to software limitations.

### Data Extraction

Data were extracted from included studies following a modified FRAME approach [34] and the GRIPP2 framework ( [35]; see Table S1). The FRAME approach was selected as it was originally developed for reporting adaptations made to psychological interventions, and it records reasons for adaptations, which is important for the present study. The GRIPP2 framework was selected as it is an evidence-based method of tracking stakeholder involvement in research. EW and AB extracted data using an extraction form created in Covidence for all included studies, then met to discuss discrepancies and reached a consensus on all data. Quality of studies is reported as percentage of studies including information from the data frameworks (e.g., type of adaptation, reason for adaptation, type of stakeholder involvement).

## Data Synthesis

The information extracted with FRAME was synthesized across all measurements to describe the adaptation processes used for anxiety and mood measures for autistic adults. A textual narrative provides a summary of the included studies. Summary information is displayed in main and supplementary tables and includes information described above. When appropriate, data extracted are categorized (e.g., type of report as self or proxy) to allow for concise summarization. See Table S1 for a full description of data extraction including the FRAME categories and GRIPP2 framework.

To answer the question of what adaptations have been made to existing measures of mental health for autistic adults (Question 1), results are presented for each measure adapted. Types of adaptations and adaptation procedures (Question 2) are summarized across all instruments to inform what adaptations are being made more generally for measuring mood and anxiety disorders in autistic adults. Available information on the involvement of autistic adults and other autism community members per each study (Question 3), extracted using the GRIPP2 framework, is included.

## Results

**Fig. 1:**
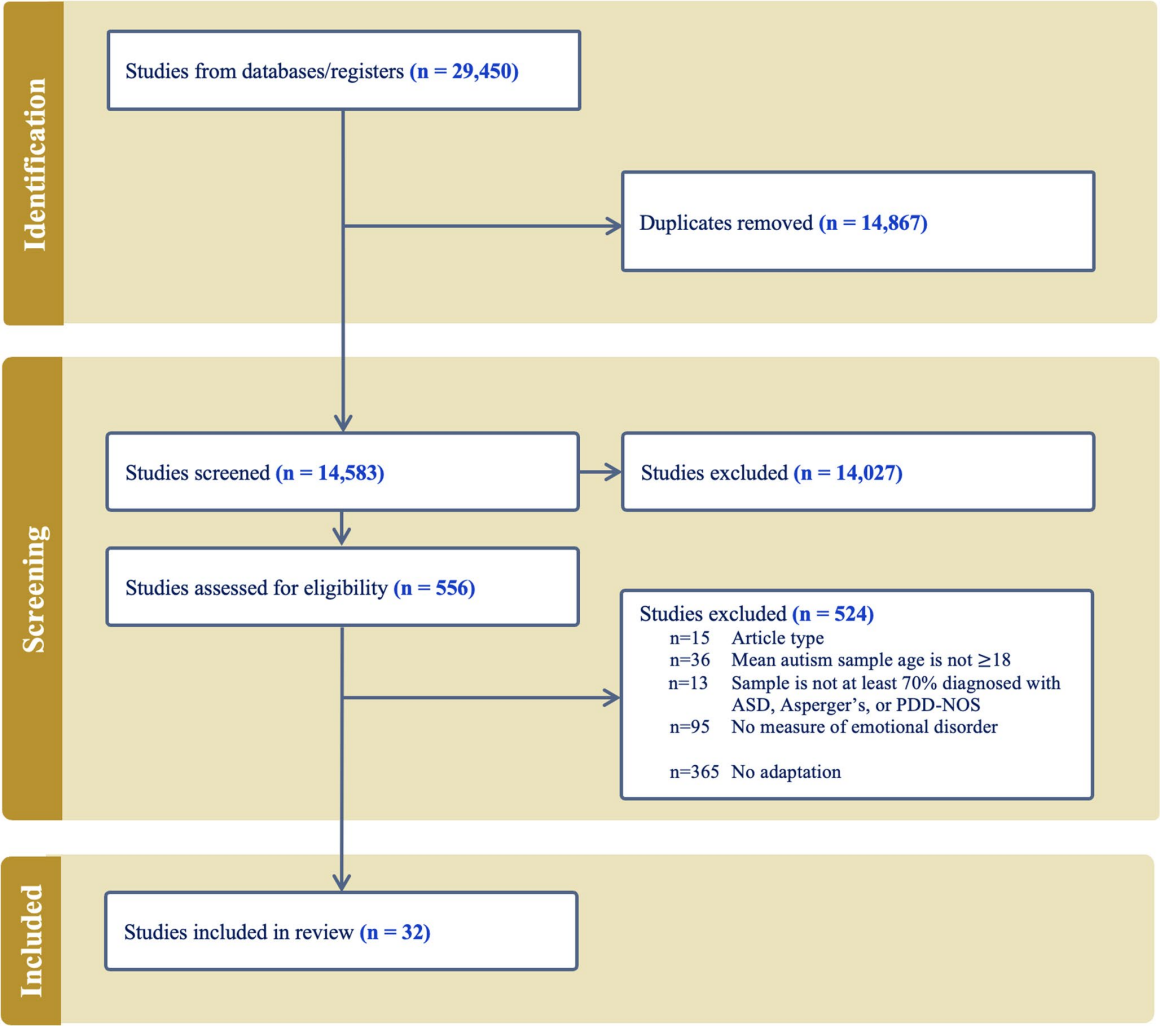
PRISMA flow diagram

From 14,583 potential studies identified in the search after removing duplicates, 14,027 were excluded based on initial screening, and a further 524 were excluded during the full-text review (Fig. 1). The 524 were excluded due: to (a) not being an included article type (e.g., meta-analysis; 2.67% of 524), (b) having a mean autism sample age < 18 years (6.87%), (c) not having a described sample that was > 70% diagnosed autistic (2.48%), (d) not including a measure of anxiety and/or mood disorders (18.13%), and f) there was no mention of adaptations to the anxiety and mood disorders measures used in the study (69.66%). Thus, of the 397 articles that met all other inclusion criteria and included a measure of anxiety and/or mood, 92% did not mention adaptations. This left 32 studies in the review (Table S3).

### Question 1 – What Adaptations Gave Been Made to Existing Measures of Anxiety and Mood for Autistic Adults?

As some studies adapted more than one measure, among the 32 studies identified, 44 measures were adapted (Table 1). Of these 44 measures, 11 had both a context and content adaptation, while 25 had only a context adaptation, and 8 had only a content adaptation.

**Table 1:** Question 1 - What adaptations have been made to existing measures of mental health for autistic adults?

Study	Measure	Summary of adaptation	What contextual adaptations were made?		What is the nature of the content adaptation?	What were the goals?		What were the reasons for the adaptation?
Schiltz et al., [77]	AMAS	Administered as proxy report, developed as self-report	informant		-	increase feasibility		cognitive capacity
Payne [48]	ASR	Survey read aloud offered/other support unspecified	format		-	increase feasibility		-
Maddox & White [54]	ADIS: Social Phobia Module	Question added about when social anxiety symptoms were most impairing. Follow-up questions added to items to ensure the behavior was due to social anxiety, rather than autism symptoms. Clinicians were given extra training to administer to an autistic person.	-		adding item(s); Other: clinicians received extra training	increase validity		Other: overlap in autism and anxiety symptoms
Payne [48]	BAI	Survey read aloud/other support unspecified	format		-	increase feasibility		-
Forbes et al. [42]	BAI	Administered as proxy report for those who could not self-report, developed as self-report	informant		-	increase feasibility		cognitive capacity
Hollocks et al. [43]	BAI	Administered as proxy report, developed as self-report	informant		-	-		cognitive capacity
Payne [48]	BDI	Participants were offered support filling out assessments in the form of reading them aloud, and potentially other forms of support.	format		-	increase feasibility		-
Forbes et al. [42]	BDI	Administered as proxy report for those who could not self-report, developed as self-report	informant		-	increase feasibility		cognitive capacity
Hollocks et al. [43]	BDI	Administered as proxy report, developed as self-report	informant		-	-		cognitive capacity
McCauley et al. [41]	BDI	Administered as proxy report, developed as self-report	informant		-	Other: be able to compare to self-report		-
Schiltz et al. [77]	BDI	Administered as proxy report, developed as self-report.	informant		-	increase feasibility		cognitive capacity
Williams et al. [51]	BDI	New norms and clinical latent scores based on autism sample	-		Other: new clinical norms for autistic sample	increase validity		Other: lack of psychometrically appropriate measures for this population
Williams et al. [38]	bMFQ	Administered to adults, developed for$\:\le\:$16 years	population		-	increase feasibility		cognitive capacity
Dieleman et al. [60]	CBCL	Administered to adults, developed for children; Generated scores out of range.	population		-	Other: have ability to compare longitudinally to child data		Other: want to compare longitudinally to child data
Dewrang & Sandberg [36]	COIS	Administered to adults, developed for children	population		-	-		-
Gotham et al. [59]	CDI-P	Items were adapted to be more relevant to the sample (e.g., changing school to work, play with to hang out with); Administered to adults, developed for children	population		refining items (wording)	increase validity; Other: be able to compare with child self-report		Other: age of sample
Gotham et al. [59]	CDRS	Items were adapted to be more relevant to the sample (e.g., changing school to work, play with to hang out with); Administered to adults, developed for children	population		refining items (wording)	increase validity; Other: be able to compare with child self-report		Other: age of sample
Ezell et al. [73]	P-ChIPS	Administered to adults, developed for children; Removed the requirement that participants recognize their fear or anxiety as irrational or excessive.	population		instructions; removing item(s)	increase validity		cognitive capacity
Russel et al. [56]	PR-CHOCI-R	Administered to adults, developed for children	population		instructions; Other: adding visual tools to depict the discomfort and anxiety basis for each OC symptoms	increase validity		Other: autism symptoms overlapping with anxiety and OCD
Dewrang & Sandberg [36]	CY-BOCS	Administered to adults, developed for children	population		-	-		-
Moss et al. [76]	CY-BOCS	Added ASD-related items, similar to CY-BOCS-PDD, but kept the obsessive section; Administered to adults, developed for children and adolescents.	population		integrating items from another measure	increase validity		-
Kildahl et al. [45]	CY-BOCS	Administered to adults, developed for children	population	-			increase validity	cognitive capacity
Lever & Geurts [74]	MINI-Plus	Wording of the MINI was adjusted to make individual items more comprehensible to individuals with autism	-		refining items (wording); breaking up items	increase validity		Other: to make more comprehensible to individuals with autism
Kildahl et al. [45]	MINI	Administered as proxy report, developed as self-report	informant	-			increase validity	cognitive capacity
Buck et al. [72]	Mini PAS-ADD	Designed for adults with ID but was administered to a broader sample of autistic adults	population		-	-		-
Kildahl et al. [45]	MADRS	Administered as proxy report, developed as self-report	informant	-			increase validity	cognitive capacity
Chew et al. [58]	PI-WSUR	Items were adapted to make more clear to the autistic population	-		refining items (wording)	increase validity		Other: overlap between anxiety and autism experiences
Shtayermmann 2007	PHQ-A	Administered to adults, developed for adolescents	population		-	-		-
Rodgers et al. [15]	PAIS	Added items regarding the experience of anxiety specific to autism (Additional items are being called the Personalised Anxiety Interview Schedule-Autism)	-		adding item(s)	increase validity		Other: overlap between autism and anxiety
Battaglia et al. [49]	SAPPA	The SAPPA was adapted (clinical research instrument) for a clinical outpatient setting by adjusting psychiatric diagnoses, making digital version, and applying to new setting	format; setting		Other: adjusted criteria for certain diagnoses	increase feasibility		Other: clinical mental health setting needs
Mazefsky et al. [71]	SADS-L	Administered as proxy report, developed as self-report	informant		-	-		cognitive capacity
Smith et al. [51]	SCARED	Reworded items to make them more appropriate for the age of the sample (e.g., “school” to “school or work”); Administered to individuals > 19 years, though the measure was developed for and validated on individuals up to 19 years	population		refining items (wording)	increase validity		Other: no measures exist to measure adults’ anxiety symptoms as reported by parents
Chew et al. [58]	SAQ	Reworded items to make them more clear to the autistic population	-		refining items (wording)	increase validity		Other: overlap between anxiety and autism experiences
Swain et al., 2015	SAS	Administered to adults, developed for adolescents	population		-	increase validity		-
Lei et al. [31]	SAS-A	Administered to adults, developed for adolescents	population		-	-		-
Pirinen et al. [50]	SPAI	Separated scores (anxiety levels) for different situations (strangers, authority figures, opposite sex, or people in general)	-		Other: formed four new separate scores for each different situation	-		-
Gillott & Standen [52]	SCAS	Items reworded to be more relevant to the target population (e.g., school changed to college/day center). Administered to adults, developed for children	population		refining items (wording)	increase validity		cognitive capacity; Other: age appropriateness (e.g., school vs. day center)
Zamzow et al. [53]	SCAS	Administered to adults, developed for children	population		-	increase validity		Other: paucity of other assessment measures for this population
Gotham et al. [59]	SCAS-p	Items reworded for relevance to the sample (e.g., changing school to work, play with to hang out with); Administered to adults, developed for children	population		refining items (wording)	increase validity; Other: be able to compare with child self-report		Other: age of sample
Joshi et al. [44]	SCID	Administered as proxy report, developed as self-report	informant		-	-		-
Russel et al. [56]	Y-BOCS	Added instructions and unspecified visual aids	population		instructions; Other: adding visual tools to depict the discomfort and anxiety basis for each OC symptoms	increase validity		Other: autism symptoms overlapping with anxiety and OCD
McDougle et al. [47]	Y-BOCS	The administration format and scoring changed categories to account for autism symptoms; Parents were present during the interview, though it is a self-report measure	informant		Other: individual items were categorized into new categories that were identified once administered to the autism sample	increase validity		cognitive capacity
Russel et al. [56]	Y-BOCS + Y-BOCS–SC	Instructions were modified to include additional definitions and an understanding check to ensure genuine obsessions and compulsions were captured	-		instructions	increase validity		Other: overlap in OCD symptoms and autism repetitive behaviors and interests
Limoges et al. [75]	YSR	Administered to adults, developed for children	population		-	-		-

#### Context adaptations

The most frequently reported context adaptation was administering a measure outside of its intended population (*n* = 20). For this adaptation, researchers administered a measure to adults when it was developed for children or adolescents [36–38].

The next most commonly reported adaptation (*n* = 12) was a change in the informant of the measure (e.g., intended to be self-report, but was completed by a a proxy). For example, the Beck Anxiety Inventory [39] and Beck Depression Inventory [40] were often administered as proxy measures to parents and caregivers, though they were developed and validated as self-report measures. One study [41] specified that this change was implemented to allow for comparison to self-report, which they also collected. However, in most cases, the reason for this adaptation was not explicit. One study did describe that the BAI and BDI were completed by parents only when the autistic adult participant could not complete it themselves, but it was not specified if this was only for people with intellectual disabilities [42]. Overall, five studies that included individuals with an intellectual disability adapted self-report instruments to be proxy-report, though they did not indicate that this was being done due to participants having an intellectual disability or out of concern regarding accessibility of the instruments [42–46]. An additional study [47] performed the Yale-Brown Obsessive Compulsive Scale (Y-BOCS) interview with both the participant and their parent present, while it was developed for the child alone.

There were four context adaptations to the format (e.g., reading aloud). The consistency of this adaptation varied; for example, one study indicated that study staff read the BDI and BAI aloud for an unspecified proportion of their sample who “needed support” [48]. Finally, one adaptation to the setting was identified (e.g., administered in a clinic but developed for research purposes; [49]).

One study performed a post-assessment adaptation to the scoring to account for the autistic experience of anxiety by separating the anxiety score by situation (strangers, authority figures, opposite sex, people in general), which was already captured by the measure but not in the scoring [50]. Additionally, Williams and colleagues [51] performed a post-assessment adaptation by generating new self-report BDI scores standardized for autistic people using two large adult samples, though this was only for the BDI as a self-report measure.

Only 25 studies included information on intellectual abilities. Of these, 12 included individuals with an intellectual disability. Among these 12 studies, six adapted self-report instruments to be proxy-report, and seven administered measures developed for use with child populations to adults or parents of adults. Of the 13 studies that only included individuals without an intellectual disability, eight used measures that were developed for children and/or adolescents, and the remaining five made adaptations to the content of the measure.

#### Content adaptations

Occasionally (*n* = 2), when researchers used child-developed measures with adults, they also indicated a content adaptation (i.e., the changing of wording to make it appropriate for the sample, such as “school” to “school or work”; [52, 53]).

Others described content adaptations that included both the changing of wording and the addition of instructions to clarify differences between features of autism and symptoms of co-occurring conditions. This most frequently occurred for measures of anxiety [54–57] and obsessive-compulsive disorder (OCD; [56, 58]). For one study, administrators received extra training to perform an anxiety interview with autistic adults [59].

### Question 2 – What Has Been the Adaptation Process?

Based on the information provided, nearly all adaptations (42/44) happened during the pre-assessment or planning phase of the identified studies (See Table 2). Across the studies, very little information was provided regarding who participated in the decision to make the adaptation. No studies specified involvement of anyone outside the study team participating in the decision-making (i.e. the decision *to make the adaptation*, as opposed to *what adaptation* to make). The sources of the adaptations (i.e., the reason that prompted the adaptation) were not directly stated, but rather implied from information presented in the background section of the manuscript. Most often clinical judgement was identified as the source (20/44), though many studies did not provide enough information to determine a source (21/44). Four studies specified previous research that prompted or justified an adaptation, while one specified that the adaptation was prompted by the need for longitudinal data [60]. Two studies indicated that both data analyses and clinical judgment prompted their adaptation [58, 61].

**Table 2:** Question 2 - What has been the adaptation process?

Schiltz et al. [77]	Measure	Summary of adaptation	When did the adaptation occur?	Were the adaptations proactive or reactive?	Were adaptations prompted by a particular source?	Who participated in the decision to adapt?
	AMAS	Administered as proxy report, developed as self-report	pre-assessment/planning	proactive	clinical judgement	researcher
Payne [48]	ASR	Survey read aloud offered/other support unspecified	-	-	clinical judgement	researcher
Maddox & White [54]	ADIS: Social Phobia Module	Question added about when social anxiety symptoms were most impairing. Follow-up questions added to items to ensure the behavior was due to social anxiety, rather than autism symptoms. Clinicians were given extra training to administer to an autistic person.	pre-assessment/planning	proactive	-	researcher
Payne [48]	BAI	Survey read aloud/other support unspecified	-	-	clinical judgement	researcher
Forbes et al., 2022	BAI	Administered as proxy report for those who could not self-report, developed as self-report	pre-assessment/planning	proactive	clinical judgement	researcher
Hollocks et al. [43]	BAI	Administered as proxy report, developed as self-report	pre-assessment/planning	proactive	-	researcher
Payne [48]	BDI	Participants were offered support filling out assessments in the form of reading them aloud, and potentially other forms of support.	-	-	clinical judgement	researcher
Forbes et al. [42]	BDI	Administered as proxy report for those who could not self-report, developed as self-report	pre-assessment/planning	proactive	clinical judgement	researcher
Hollocks et al. [22]	BDI	Administered as proxy report, developed as self-report	pre-assessment/planning	proactive	-	researcher
McCauley et al. [41]	BDI	Administered as proxy report, developed as self-report	pre-assessment/planning	proactive	-	researcher
Schiltz et al., 2023	BDI	Administered as proxy report, developed as self-report.	pre-assessment/planning	proactive	clinical judgement	researcher
Williams et al. [51]	BDI	New norms and clinical latent scores based on autism sample	post assessment	proactive	data analyses	researcher
Williams et al. [38]	bMFQ	Administered to adults, developed for$\:\le\:$16 years	pre-assessment/planning	proactive	-	researcher
Dieleman et al. [60]	CBCL	Administered to adults, developed for children; Generated scores out of range.	pre-assessment/planning	proactive	Other: need for longitudinal comparison and previous studies finding it an adequate measure to use with young adults	researcher
Dewrang & Sandberg [36]	COIS	Administered to adults, developed for children	pre-assessment/planning	proactive	-	researcher
Gotham et al. [59]	CDI-P	Items were adapted to be more relevant to the sample (e.g., changing school to work, play with to hang out with); Administered to adults, developed for children	pre-assessment/planning	proactive	clinical judgement	researcher
Gotham et al. [59]	CDRS	Items were adapted to be more relevant to the sample (e.g., changing school to work, play with to hang out with); Administered to adults, developed for children	pre-assessment/planning	proactive	clinical judgement	researcher
Ezell et al. [73]	P-ChIPS	Administered to adults, developed for children; Removed the requirement that participants recognize their fear or anxiety as irrational or excessive.	pre-assessment/planning	proactive	clinical judgement	researcher
Russel et al. [56]	PR-CHOCI-R	Administered to adults, developed for children	pre-assessment/planning	proactive	-	researcher
Dewrang & Sandberg [36]	CY-BOCS	Administered to adults, developed for children	pre-assessment/planning	proactive	-	researcher
Moss et al. [76]	CY-BOCS	Added ASD-related items, similar to CY-BOCS-PDD, but kept the obsessive section; Administered to adults, developed for children and adolescents.	pre-assessment/planning	proactive	-	researcher
Kildahl et al. [45]	CY-BOCS	Administered to adults, developed for children	pre-assessment/planning	proactive	clinical judgement	researcher
Lever & Geurts [74]	MINI-Plus	Wording of the MINI was adjusted to make individual items more comprehensible to individuals with autism	pre-assessment/planning	proactive	clinical judgement	researcher
Kildahl et al. [45]	MINI	Administered as proxy report, developed as self-report	pre-assessment/planning	proactive	clinical judgement	researcher
Buck et al. [72]	Mini PAS-ADD	Designed for adults with ID but was administered to a broader sample of autistic adults	pre-assessment/planning	-	-	researcher
Kildahl et al. [45]	MADRS	Administered as proxy report, developed as self-report	pre-assessment/planning	proactive	clinical judgement	researcher
Chew et al. [58]	PI-WSUR	Items were adapted to make more clear to the autistic population	pre-assessment/planning	proactive	data analyses; clinical judgement	researcher
Shtayermmann, 2007	PHQ-A	Administered to adults, developed for adolescents	pre-assessment/planning	proactive	-	researcher
Rodgers et al. [55]	PAIS	Added items regarding the experience of anxiety specific to autism (Additional items are being called the Personalised Anxiety Interview Schedule-Autism)	pre-assessment/planning	proactive	-	researcher
Battaglia et al. [49]	SAPPA	The SAPPA was adapted (clinical research instrument) for a clinical outpatient setting by adjusting psychiatric diagnoses, making digital version, and applying to new setting	pre-assessment/planning	proactive	-	researcher
Mazefsky et al. [71]	SADS-L	Administered as proxy report, developed as self-report	pre-assessment/planning	proactive	-	researcher
Smith et al. [57]	SCARED	Reworded items to make them more appropriate for the age of the sample (e.g., “school” to “school or work”); Administered to individuals > 19 years, though the measure was developed for and validated on individuals up to 19 years	pre-assessment/planning	proactive	-	researcher
Chew et al. [58]	SAQ	Reworded items to make them more clear to the autistic population	pre-assessment/planning	proactive	data analyses; clinical judgement	researcher
Swain et al. [78]	SAS	Administered to adults, developed for adolescents	pre-assessment/planning	proactive	-	researcher
Lei et al. [31]	SAS-A	Administered to adults, developed for adolescents	pre-assessment/planning	proactive	-	researcher
Pirinen et al. [50]	SPAI	Separated scores (anxiety levels) for different situations (strangers, authority figures, opposite sex, or people in general)	post assessment	-	-	researcher
Gillott & Standen [52]	SCAS	Items reworded to be more relevant to the target population (e.g., school changed to college/day center). Administered to adults, developed for children	pre-assessment/planning	proactive	clinical judgement	researcher
Zamzow et al. [53]	SCAS	Administered to adults, developed for children	pre-assessment/planning	proactive	-	researcher
Gotham et al. [59]	SCAS-p	Items reworded for relevance to the sample (e.g., changing school to work, play with to hang out with); Administered to adults, developed for children	pre-assessment/planning	proactive	clinical judgement	researcher
Joshi et al. [44]	SCID	Administered as proxy report, developed as self-report	pre-assessment/planning	proactive	-	researcher
Russel et al. [56]	Y-BOCS	Added instructions and unspecified visual aids	pre-assessment/planning	proactive	-	researcher
McDougle et al. [47]	Y-BOCS	The administration format and scoring changed categories to account for autism symptoms; Parents were present during the interview, though it is a self-report measure	pre-assessment/planning	proactive	clinical judgement	researcher
Russel et al. [56]	Y-BOCS + Y-BOCS–SC	Instructions were modified to include additional definitions and an understanding check to ensure genuine obsessions and compulsions were captured	pre-assessment/planning	proactive	data analyses; clinical judgement	researcher
Limoges et al. [75]	YSR	Administered to adults, developed for children	pre-assessment/planning	proactive	-	researcher

Regarding the post-assessment process, 11 of the 25 studies reported some kind of psychometric information on the reliability of the measure post-adaptations. The majority (*n* = 6) of these reported internal consistency only (Cronbach’s alpha). The remainder (*n* = 5) reported interrater agreement on interview administration. Three of the 25 studies reported some kind of information on the validity of the adapted measure (one describing convergent validity with community diagnosis and two describing sensitivity and specificity analyses).

### Question 3 - How Were Autistic Adults Involved?

Of the 32 studies included, only one described the involvement of autistic adults in the adaptation process. Chew and colleagues [58] adequately described their involvement of autistic adults in the adaptation process, based on the GRIPP2. Specifically, they included autistic adults with the aim of accurately capturing the autistic experience of social anxiety and OCD using the Social Anxiety Questionnaire (SAQ; [62]) and the Padua Inventory for OCD (PI-WSUR; [63, 64]). Authors indicated that two autistic consultants and two psychiatrists with experience working with autistic adults with co-occurring mental health challenges were presented the original SAQ and PI-WSUR items, asked if they were ambiguous, and provided suggestions to modify ambiguous items to clarify them. These individuals were later invited to provide further comments and edit the proposed clarifications via email before they were finalized. Overall, the consultants identified several items they believed would benefit from further clarification. The authors commented that one limitation to this involvement is that all consultants were highly educated, which may have influenced their feedback.

## Discussion

Research on mood and anxiety disorders in autistic adults is rapidly proliferating and concerns have been raised regarding the measurement of these conditions in autistic adults [1, 4, 65]. Specifically, concerns have been raised regarding the clinical utility and psychometric validity of tools developed for and normed on allistic individuals. Somewhat surprisingly, our review found that, of 365 studies including mood or anxiety disorder measures published over the past 30 years, 92% did not identify any adaptation. In the remaining 32 studies, 44 different adaptations (Table S2) were reported. Notably, however, less than half of the studies provided explicit justification for adaptation, and even fewer described the adaptation process. Despite increasing calls for community-based participatory research and inclusion of autistic perspectives in measure adaptation, only one study was identified to have included autistic individuals in the adaptation process [58] (not accounting for studies authored by autistic researchers who did not explicitly indicate as such).

Autism researchers and clinicians periodically make measure adaptations (e.g., extra training on interview administration to autistic adults, adding follow-up questions to interviews to clarify mental health symptoms from autism traits, reading aloud to participants), yet these are not systematically documented. Lack of careful documentation impedes our ability to explore the effects that changes have on the psychometric properties of the instruments when used with autistic people. Of the 44 adaptations identified in the current review, only 16 were accompanied by some information on the adapted measure’s reliability or validity (Table S2). The majority of studies were limited to reporting of internal consistency reported as Cronbach’s alpha (*n* = 6) and interrater agreement (*n* = 5).

Overall, there was a lack of information included regarding the adaptation processes. One exception was a study by Chew et al. [58], using the Padua Inventory for OCD and Social Anxiety Questionnaire. In their paper, they specify and describe individual items they changed. Subsequently, they compare results from each measure in its adapted and original forms to understand how the adaptations affect the measure. While the latter procedure may not be feasible for every study, descriptions of all adaptations and a brief justification as to why they were made should be a requirement of all studies. Such information is critical to interpretation of results, as well as replicability across studies. Information gained by comparing adapted to non-adapted measures informs future decisions regarding the measurement of mood and anxiety disorders and the use of adapted measures.

Studies that adapted self-report measures to be proxy-report tended to include individuals with an intellectual disability, and the reasons for such an adaptation were determined to be clinical judgement (Table 2). It is not clear, however, what the specific justification is. For example, the clinician’s impression may be that the available measures are not adequate/accessible for autistic people with an intellectual disability. The clinician could also be making assumptions about those individuals’ capacity for self-report of mood and anxiety symptoms. Given the prevalent belief that every individual with an intellectual disability cannot read or understand nuanced topics, along with the current lack of measures with accessible language, it may be that these adaptations are made due to researchers’ own biases. A recent systematic review found mixed agreement between self- and proxy-report among those with an intellectual disability (not autism-specific; [66]). Within this review, one study found that adults with a mild intellectual disability report higher levels of affective- and cognitive-based depression symptoms, while informants (staff) report higher frequency of somatic-based symptoms [67]. A second study found moderate correlations between staff- and self-report on a depression measure [68]. Clearly more research is needed to develop accessible self-report measures and to understand what is driving differences in self- and proxy-report to inform assessment of mood and anxiety symptoms in those who cannot provide self-report.

This review found that the most common context adaptation for those without an intellectual disability was administering a child/adolescent measure to an adult. Several of these studies included individuals under 18 years of age, which may be seen as some justification for this decision. However, across the studies, there was a lack of information provided on the decision-making process behind this adaptation. One study justified using the Child Behavior Checklist with adults to allow comparisons to earlier administrations collected in childhood [60]. However, they did not describe how they scored for adults outside of the age standardization. The remaining studies did not provide information as to why child/adolescent measures were administered to adults. Other than this example, no studies provided explicit justification for administering child/adolescent measures to adults.

In the future, when describing adaptations to existing measures, studies should include a description of how autistic adults were involved in the process. The modified FRAME approach used by this review provides a structure to inform how adaptation and report such information. This approach could be utilized by future studies measuring the anxiety and mood disorders in autistic adults. Further, psychometric analyses need to be reported following adaptations to understand the validity of the changed measure. If no adaptations were made, future studies should specify this (e.g., “The BDI-II was used without modification”). Similarly, the GRIPP2 framework should be referenced to provide structure in clear reporting of stakeholder involvement.

Importantly, although our review focused on adaptations to existing measures, several established instruments have demonstrated robust psychometric performance in autistic adult samples even without major modification. For instance, traditional measures such as the Beck Depression Inventory–II have shown relevant factor structures and adequate validity when used with autistic adults [51, 59]. These findings suggest that traditional conceptualizations of mental-health constructs and the measures based on these conceptualizations may remain applicable, provided that language, context, and administration procedures are adapted thoughtfully when needed. Conversely, the largely undocumented adaptations identified in our review make it difficult to draw firm conclusions about which modifications improve versus compromise validity.

It is important to note that there are measures of anxiety and mood disorders developed specifically for autistic adults and therefore not captured in this review, as this was not considered an *adaptation*. The Anxiety Scale for Autism-Adults [69] and Autistic Depression Assessment Tool–Adult [2], for example, are measures that were developed based on autistic people’s experience and expression of anxiety and depression. Similarly, other instruments capturing transdiagnostic constructs (e.g., Emotional Dysregulation Inventory, Aberrant Behavior Checklist) have been developed and/or validated with autistic samples [28, 70, 71]. While these measures did not meet criteria for inclusion in the present review, several were developed to address the lack of validated and appropriate measures for autistic adults. The processes of developing these measures may provide valuable information on the context and content adaptations that should be considered for existing measures, as well as provide guidelines for documenting the adaptation process.

Looking forward, researchers conducting studies now should explicitly state whether adaptations were made (e.g., “The BDI-II was used without modification”), provide detailed descriptions and justifications for any changes, and report psychometric analyses following adaptation. It is notable that prevailing scientific and publishing practice (e.g., space constraints, norms that privilege “standard” measures, and concerns that adaptations may be viewed as methodological weakness) may inadvertently discourage such transparency. These barriers could be mitigated through clearer journal guidance endorsing the reporting of adaptations, the routine use of supplementary materials for detailed methodological documentation, and reviewer training that frames transparent reporting as a marker of rigor rather. Further, when feasible, autistic adults should be active partners in decision-making regarding adaptation. Future work should prioritize the development of accessible self-report tools, evaluation of when proxy-report is warranted, and systematic comparisons of adapted and non-adapted versions of measures to determine which modifications maintain or improve validity. There is also a need to identify which traditional measures retain robustness for autistic adults and under what conditions, as well as to refine adaptations that enhance accessibility without altering the construct being measured.

The findings of this review should be interpreted within the context of its strengths and limitations. To our knowledge, this is the only review of adaptations made to anxiety and mood disorder measures used with autistic adults. Although the search strategy employed was broad, by design, to capture all studies using such measures, there is always a risk of missing pertinent studies. Given that 92% of studies meeting all other inclusion criteria had a measure of mood and/or anxiety yet did not mention any form of adaptation, it is possible that adaptations simply were not documented (although this is a limitation of the reviewed research) and therefore not captured here.

## Conclusion

This is the first review to systematically describe the adaptations made to measures of anxiety and mood disorders used with autistic adults. The review found that: (1) adaptations to measures of anxiety and mood used with autistic adults are not well-documented, (2) those that are documented include sparse detail on the reasons for the adaptation and the adaptation process, and (3) there has been very little explicit involvement of the autistic community in the decision or process to adapt measures. Notably, the two most frequently implemented adaptations are the administration of a measure developed for children to adults and the administration of a measure developed for self-report to a proxy. As information provided on reasons for adaptations was lacking, there is concern that these changes are based on assumptions or biases about autistic adults. Future research should systematically report if adaptations were made to anxiety and mood disorder measures used with autistic adults, and, in the cases where adaptations were made, specific information about what changes were employed and the psychometric properties of the adapted measures. Explicit justifications for adaptations should be provided, and, whenever possible, autistic adults should be included in the adaptation process, in order to inform best practices of the application of these measures.

## Key References

1. M. J. Hollocks, J. W. Lerh, I. Magiati, R. Meiser-Stedman, and T. S. Brugha, “Anxiety and depression in adults with autism spectrum disorder: a systematic review and meta-analysis,” *Psychol. Med.*, vol. 49, no. 4, pp. 559–572, Mar. 2019, doi: 10.1017/S0033291718002283.
  This comprehensive systematic review and meta-analysis offers valuable insights into the high prevalence of anxiety and depression in adults with autism spectrum disorder and underscoring the urgent need for improved assessment and mental health support in this population.
2. S. A. Cassidy, L. Bradley, E. Bowen, S. Wigham, and J. Rodgers, “Measurement properties of tools used to assess depression in adults with and without autism spectrum conditions: A systematic review: Measurement Properties of Depression Tools,” *Autism Res.*, vol. 11, no. 5, pp. 738–754, May 2018, doi: 10.1002/aur.1922.
  A systematic review that provides an evaluation of depression assessment tools, highlighting crucial limitations and the need for measures either made for autism, adapted for autism, and/or validated in autistic samples in order to ensure accurate diagnosis and support for autistic adults.
3. X. Y. Chew, D. Leong, K. M. Khor, G. M. Y. Tan, K.-C. Wei, and I. Magiati, “Clarifying Self-Report Measures of Social Anxiety and Obsessive-Compulsive Disorder to Improve Reporting for Autistic Adults,” *Autism Adulthood*, vol. 3, no. 2, pp. 129–146, Jun. 2021, doi: 10.1089/aut.2019.0064
  The sole article found in the systematic review that described the inclusion of autistic people in their adaptation methodology. The article demonstrates that while standard social anxiety questionnaires work well as-is, obsessive-compulsive measures benefit greatly from simple clarifications to ensure accurate, autism-sensitive symptom reporting.

## Supplementary Information

Below is the link to the electronic supplementary material.

Supplementary Material 1

## Data Availability

No datasets were generated or analysed during the current study.
