# Supplementary material for: Adapting Measures of Anxiety and Mood Disorders for Use with Autistic Adults: A Systematic Review
Source: Curr Dev Disord Rep. 2026 Mar 16;13(1):4. doi: 10.1007/s40474-026-00348-3 (PMC12992341; doi:10.1007/s40474-026-00348-3)
Supplement: Supplementary file 1 — Supplementary Material 1 [file 40474_2026_348_MOESM1_ESM.docx]

**Table S1.** **Data extraction form**

| **Category** | **Variable** | **Response options** |
| --- | --- | --- |
| Article characteristics | Title |  |
|  | First Author |  |
|  | Year of Publication |  |
|  | Design | Case study, observational (1 group), observational (comparison group), experimental, other |
|  | Summary of adaptation |  |
| Autism sample characteristics | Participants’ age (mean & standard deviation) |  |
|  | Cognitive ability | ID, non-ID, not specified |
|  | Percent female |  |
|  | Diagnoses for entire autism sample |  |
|  | Diagnoses for any of autism sample |  |
|  | Percent non-white |  |
|  | Percent Hispanic |  |
|  | Describe income level |  |
|  | Describe education level |  |
| Adapted measure characteristics | Measure name(s) |  |
|  | Method(s) of administration | Survey, interview, not specified |
|  | Type of report | Self, proxy |
|  | Domains measured |  |
|  | Reliability analyses and results |  |
|  | Validity analyses and results |  |
| FRAME information | |  |
| *Process* | When did the adaptation occur? | Pre-assessment/planning, during assessment, post-assessment, not specified, other |
|  | Were adaptations planned or reactive? | Proactive, reactive, not specified, other |
|  | Were adaptations prompted by a particular source (e.g. data analyses)? | Data analyses, clinical judgement, not specified, other |
|  | Who participated in the decision to adapt? | Measure developer, researcher, individual administrator, other professional, other community member, autistic person, not specified, other |
|  | What was adapted? | Item content, instructions, administration, other |
|  | What contextual adaptations were made? | Format, setting, personnel, population, informant, not specified, none, other |
|  | What is the nature of the content adaptation? | Refining items (wording), instructions, adding item(s), removing item(s), substituting items, reordering items, breaking up items, integrating items from another measure, repeating items, not specified, other |
| *Goal* | What were the goals? | Increase feasibility, increase validity, address cultural factors, not specified, other |
|  | What were the reasons for the adaptation? | Race/ethnicity, gender identity, sexual orientation, access to resources, cognitive capacity, physical capacity, literacy/education level, knowledge of English, cultural/religious norms, comorbidity, motivation, not specified, other |
| GRIPP2 information | |  |
| *Aim* | What is the aim of patient and public involvement (PPI) in the study? |  |
| *Methods* | Describe the methods used for PPI in the study. |  |
| *Study results* | Report the results of the PPI in the study, including both negative and positive outcomes. |  |
| *Discussion and conclusions* | Comment on the extent to which PPI influenced the study overall, per authors’ description. Describe positive and negative effects, per authors’ description. |  |
| *Reflections/critical perspective* | Comment on the study, reflecting on the things that the authors describe as going well and those that did not. |  |

**Table S2. Overview of Adaptations to Anxiety and Mood Disorder Measures, Organized by Measure**

| Study | Name of adapted measure |  | Summary of adaptation | Format | Type of report | Reliability analyses and results | Validity analyses and results | |  |
| --- | --- | --- | --- | --- | --- | --- | --- | --- | --- |
| Schiltz et al., 2023 | Adult Manifest Anxiety Scale | AMAS | Administered as proxy report, developed as self-report | survey | informant | T1 α =.90;  T2 α =.89 | - | |  |
| Payne, 2016 | Adult Self Report | ASR | Survey read aloud offered /other support unspecified | survey; interview | self | - | - | |  |
| Maddox & White, 2015 | Anxiety Disorders Interview Schedule for DSM-IV: Social Phobia Module | ADIS: Social Phobia Module | Question added about when social anxiety symptoms were most impairing. Follow-up questions added to items to ensure the behavior was due to social anxiety, rather than autism symptoms. Clinicians were given extra training to administer to an autistic person. | interview | self | - | - | |  |
| Payne, 2016 | Beck Anxiety Inventory | BAI | Survey read aloud/other support unspecified | survey; interview | self | - | - | |  |
| Forbes et al., 2022 | Beck Anxiety Inventory | BAI | Administered as proxy report for those who could not self-report, developed as self-report | survey | informant | - | - | |  |
| Hollocks et al., 2022 | Beck Anxiety inventory | BAI | Administered as proxy report, developed as self-report | survey | informant | - | - | |  |
| Payne, 2016 | Beck Depression Inventory | BDI | Participants were offered support filling out assessments in the form of reading them aloud, and potentially other forms of support. | survey; interview | self | - | - | |  |
| Forbes et al., 2022 | Beck Depression Inventory | BDI | Administered as proxy report for those who could not self-report, developed as self-report | survey | informant | - | - | |  |
| Hollocks et al., 2022 | Beck Depression inventory | BDI | Administered as proxy report, developed as self-report | survey | informant | - | - | |  |
| McCauley et al., 2020 | Beck Depression Inventory | BDI | Administered as proxy report, developed as self-report | survey | informant | - | - | |  |
| Schiltz et al., 2023 | Beck Depression Inventory | BDI | Administered as proxy report, developed as self-report. | survey | informant | T1 α =.92;  T2 α =.93 | - | |  |
| Williams et al., 2021 | Beck Depression Inventory | BDI | New norms and clinical latent scores based on autism sample | survey | self | α =.895 | ROC analyses, fair discrimination between groups; strong positive correlation with GAD-7 and QoL | |  |
| Williams et al., 2022 | Brief Mood and Feelings Questionnaire | bMFQ | Administered to adults, developed for $\leq$16 years | survey | self | α =.81 | - | |  |
| Dieleman et al., 2017 | Child Behavior Checklist (ASEBA) | CBCL | Administered to adults, developed for children; Generated scores out of range. | survey | informant | α =.92 for internalizing and externalizing disorder subscales | - | |  |
| Dewrang & Sandberg, 2011 | Child Obsessive Compulsive Impact Scale | COIS | Administered to adults, developed for children | survey | informant | - | - | |  |
| Gotham et al., 2015 | Children's Depression Inventory, Parent-Rated Version | CDI-P | Items were adapted to be more relevant to the sample (e.g., changing school to work, play with to hang out with); Administered to adults, developed for children | survey | informant | α=0.73; Self and proxy report were associated, r=0.67 for CDI Functional scale; Self and informants were not associated for CDI Emotional scale | - | |  |
| Gotham et al., 2015 | Children's Depression Rating Scale | CDRS | Items were adapted to be more relevant to the sample (e.g., changing school to work, play with to hang out with); Administered to adults, developed for children | interview | informant | α=0.85; Self and proxy report were associated r=.47 to .76 | - | |  |
| Ezell et al., 2019 | Children's Interview for Psychiatric Symptoms-Parent Version | P-ChIPS | Administered to adults, developed for children; Removed the requirement that participants recognize their fear or anxiety as irrational or excessive. | interview | informant | Interrater agreement = 100% | - | |  |
| Russel et al., 2013 | Children's Obsessive Compulsive Inventory-Parent Version | PR-CHOCI-R | Administered to adults, developed for children | interview | informant | - | - | |  |
| Dewrang & Sandberg, 2011 | Children's Yale-Brown Obsessive Compulsive Scale | CY-BOCS | Administered to adults, developed for children | interview | informant | Interrater reliability obsessive (rho=.683, n=13, p<.01), compulsive (rho=.906, n=13, p<.01), overall (rho=.941, n=6, p<.01) | - | |  |
| Moss et al., 2015 | Children's Yale-Brown Obsessive Compulsive Scale | CY-BOCS | Added ASD-related items, similar to CY-BOCS-PDD, but kept the obsessive section; Administered to adults, developed for children and adolescents. | interview | self | - | - | |  |
| Kildahl et al., 2019 | Children's Yale-Brown Obsessive-Compulsive Scale | CY-BOCS | Administered to adults, developed for children | interview | informant | - | - | |  |
| Lever & Geurts, 2016 | Mini International Neuropsychiatric Interview Plus | MINI-Plus | Wording of the MINI was adjusted to make individual items more comprehensible to individuals with autism | interview | self | - | - | |  |
| Kildahl et al., 2019 | Mini Neuropsychiatric Interview | MINI | Administered as proxy report, developed as self-report | interview | informant | - | - | |  |
| Buck et al., 2014 | Mini Psychiatric Assessment Schedule for Adults with Developmental Disability | Mini PAS-ADD | Designed for adults with ID but was administered to a broader sample of autistic adults | interview | informant | - | Diagnoses of depression (p=.001)and OCD (p=.008) associated (x^2^=16.63) with parent-reported community diagnoses | |  |
| Kildahl et al., 2019 | Montgomery-Asberg Depression Rating Scale | MADRS | Administered as proxy report, developed as self-report | interview | informant | - | - | |  |
| Chew et al., 2021 | Padua Inventory for OCD | PI-WSUR | Items were adapted to make more clear to the autistic population | survey | self | α=.96 | - | |  |
| Shtayermmann, 2007 | Patient Health Questionnaire for Adolescents | PHQ-A | Administered to adults, developed for adolescents | survey | self | - | MDD domain: 73% sensitivity; 98% specificity. GAD domain: 50% sensitivity, 98% specificity | |  |
| Rodgers et al., 2024 | Personalized Anxiety Interview Schedule | PAIS | Added items regarding the experience of anxiety specific to autism (Additional items are being called the Personalised Anxiety Interview Schedule-Autism) | interview | self | ICC = .817 | - | |  |
| Battaglia et al., 2016 | Schedule for the Assessment of Psychiatric Problems Associated with Autism | SAPPA | The SAPPA was adapted (clinical research instrument) for a clinical outpatient setting by adjusting psychiatric diagnoses, making digital version, and applying to new setting | interview | self; informant | - | - | |  |
| Mazefsky et al., 2008 | Schedule of Affective Disorders and Schizophrenia-Lifetime Version | SADS-L | Administered as proxy report, developed as self-report | interview | informant | - | - | |  |
| Smith et al., 2019 | Screen for anxiety and related emotional disorders | SCARED | Reworded items to make them more appropriate for the age of the sample (e.g., "school" to "school or work"); Administered to individuals >19 years, though the measure was developed for and validated on individuals up to 19 years | survey | self; informant | Parent report internal consistency, α=.93; Self report internal consistency α=.96 | - | |  |
| Chew et al., 2021 | Social Anxiety Questionnaire | SAQ | Reworded items to make them more clear to the autistic population | survey | self | α=.92 | - | |  |
| Swain et al., 2015 | Social Anxiety Scale | SAS | Administered to adults, developed for adolescents | survey | self; informant | - | - | |  |
| Lei et al., 2020 | Social Anxiety Scale: Adolescents | SAS-A | Administered to adults, developed for adolescents | survey | self | - | - | |  |
| Pirinen et al., 2024 | Social Phobia and Anxiety Inventory | SPAI | Separated scores (anxiety levels) for different situations (strangers, authority figures, opposite sex, or people in general) | survey | self | - | - | |  |
| Gillott & Standen, 2007 | Spence Children's Anxiety Scale | SCAS | Items reworded to be more relevant to the target population (e.g., school changed to college/day center). Administered to adults, developed for children | survey | informant | - | - | |  |
| Zamzow et al., 2016 | Spence Children's Anxiety Scale | SCAS | Administered to adults, developed for children | survey | self | - | - | |  |
| Gotham et al., 2015 | Spence Children's Anxiety Scale-Parent | SCAS-p | Items reworded for relevance to the sample (e.g., changing school to work, play with to hang out with); Administered to adults, developed for children | survey | informant |  | | - | |
| Joshi et al., 2013 | Structured Clinical Interview for DSM-5 | SCID | Administered as proxy report, developed as self-report | interview | self; informant | Agreement on diagnostic coding, kappa=.98; Reliability of diagnostic review process, kappa=.87 | - | |  |
| Russel et al., 2013 | Yale Brown Obsessive Compulsive Scale-Symptom Checklist | Y-BOCS | Added instructions and unspecified visual aids | survey | self | - | - | |  |
| McDougle et al., 1995 | Yale-Brown Obsessive Compulsive Scale | Y-BOCS | The administration format and scoring changed categories to account for autism symptoms; Parents were present during the interview, though it is a self-report measure | interview | self; informant | - |  | |  |
| Russel et al., 2005 | Yale-Brown Obsessive-Compulsive Scale and Ancillary Symptom Checklist | Y-BOCS + Y-BOCS–SC | Instructions were modified to include additional definitions and an understanding check to ensure genuine obsessions and compulsions were captured | interview | self | High level of interrater agreement (value not reported) | - | |  |
| Limoges et al., 2005 | Youth Self-Report (ASEBA) | YSR | Administered to adults, developed for children | survey | self | - | - | |  |

**Table S3.** **Study Details**

| Study | N | Mean age (SD) [Range] | Cognitive ability | Percent female | Other diagnoses -entire group | Other diagnoses specified for anyone in autism group | Percent non-white | Percent Hispanic | Income level | Education level | GRIPP Information Reported |
| --- | --- | --- | --- | --- | --- | --- | --- | --- | --- | --- | --- |
| Battaglia et al., 2016 | 116 | 27.1 (10.5)  [18-58] | - | 16.38 | none | impulse control disorder, anxiety, OCD, MDD, ADHD, tics, psychosis, bipolar, sexual disorder, phobia, schizophrenia, eating disorder | 41 | 17 | - | - | No |
| Buck et al., 2014 | 89 | 36.3 (6.1)  [26-50] | Non-ID; ID | 28.8 | - | ID, seizure disorder, depression, mania, anxiety, OCD, psychotic disorder | - | - | - | - | No |
| Chew et al., 2021 | 50 | - | - | 42 | - | ADHD, SLD, depression, anxiety, OCD, eating disorder, other not specified | 52 | - | - | - | Yes |
| Dewrang & Sandberg, 2011 | 27 | 18.52 (2.21)  [14-24] | Non-ID | 18.52 | - | - | - | - | - | - | No |
| Dieleman et al., 2017 | 114 | 19.0 (2.3)  [14-23] | Non-ID; ID | 21.05 | - | ADHD, SLD, motor disabilities, other unspecified | - | - | - | 17.5 higher education, 26.3 special secondary education, 28.9 regular secondary education, .9 special primary education | No |
| Ezell et al., 2019 | 20 | 18.94 (2.20)  [15-22] | Non-ID; ID | 0 | - | GAD, specific phobia, social phobia | - | - | - | - | No |
| Forbes et al., 2022 | 121 | 23.0 (22.5-23.7) - median (IQR) | Non-ID; ID | 12.4 | - | - | - | - | - | - | No |
| Gillott & Standen, 2007 | 34 | 37  [18-56] | ID | 32.35 | ID | - | - | - | - | - | No |
| Gotham et al., 2015 | 50 | 20.7 (3.9)  [16-31] | Non-ID | 10 | - | MDD, dysthymic disorder, mood disorder-NOS, GAD, OCD, anxiety disorder-NOS | 18 | 0 | - | - | No |
| Hollocks et al., 2022 | 81 | 23.2 (.79)  [21-25] | Non-ID; ID | 8.64 | none | - | - | - | - | - | No |
| Joshi et al., 2013 | 63 | 29.2 (11.0)  [18-63] | Non-ID; ID | 35 | none | Tic disorder, Tourette's disorder, ADHD, ODD, conduct disorder, antisocial personality disorder, MDD, bipolar, psychosis, specific phobia, separation anxiety disorder, agoraphobia, generalized anxiety, social phobia, OCD, panic disorder, PTSD, substance use disorders, alcohol use/dependence, drug abuse/ dependence, enuresis, encopresis | 5 | - | Socioeconomic status (FFISS) 2.1 ¬± 0.9 (from subset of 36 people) | 0% less than high school, 44% high school, 44% college, 12% graduate school | No |
| Kildahl et al., 2019 | 2 | 26  [22-30] | ID | 0 | ID, congenital blindness, anxiety | OCD, major depressive episode | - | - | - | - | No |
| Lei et al., 2020 | 21 | 18.33 (.48)  [17-19] | Non-ID | 47.6 | - | anxiety, depression, ADHD, sensory processing disorder, dyspraxia | 4.8 | - | - | currently attending college | No |
| Lever & Geurts, 2016 | 138 | 46.5  [19-79] | Non-ID | 30.43 | - | depression, dysthymia, MPDD, panic disorder, agoraphobia, social phobia, specific phobia, PTSD, OCD, GAD, substance-related disorders, eating disorders, somatoform disorders, ADHD, conduct disorder | - | - | - | .72% low education, 31.16% middle education, 68.12% high education | No |
| Limoges et al., 2005 | 27 | 21.1 (3.6)  [16-27] | Non-ID | 7.4 | none | ADHD, expressive language disorder | - | - | - | - | No |
| Maddox & White, 2015 | 28 | 23.93 (6.92)  [16-45] | Non-ID | 46.4 | - | SAD | 21.4 | 3.6 | - | - | No |
| Mazefsky et al., 2008 | 17 | 21.2 (4.5)  [18-32] | Non-ID; ID | 6 | none | GAD, specific phobia, MDD, minor depressive disorder, bipolar | - | - | - | - | No |
| McCauley et al., 2020 | 150 | 25.73  [18-27] | - | 21 | - | ADHD, anxiety, depression | 24 | - | - |  | No |
| McDougle et al., 1995 | 50 | 30.4 (7.9) | Non-ID; ID | 28 | - | OCD | - | - | - | - | No |
| Moss et al., 2015 | 58 | 43.3 (9.1)  [29-64] | Non-ID; ID | 17.24 | - | OCD, depression, bipolar, anxiety | - | - | - | - | No |
| Payne, 2016 | 23 | 22.9 (4.1) | - | 31.8 | - | - | 13.7 | 4.5 | - | - | No |
| Pirinen et al., 2024 | 34 | 23.7 (3.2)  [19-33] | Non-ID | 21.88 | - | - | - | - | - | - | No |
| Rodgers et al., 2024 | 34 | 39.5 (15.3), experimental; 33.1 (10.2), control  [18-62] | - | 32.35 | anxiety | GAD, SAD, specific phobia, separation anxiety, panic disorder, agoraphobia, depression, OCD, body dysmorphic disorder, PTSD, somatic symptom disorder | 5.88 | - | - | - | No |
| Russel et al., 2005 | 40 | 27.9 (8.5) | Non-ID | 15 | - | OCD, affective disorder, schizophrenia, anxiety | - | - | - | - | No |
| Russel et al., 2013 | 46 | 26.9  [14-65] | Non-ID | 23.91 | OCD | - | - | - | - | - | No |
| Schiltz et al., 2023 | 130 | Time 1: 20.15 (1.68); Time 2: 26.67 (1.75) | - | 22.31 | - | - | 17.69 | 3.85 | - |  | No |
| Shtayermman, 2007 | 10 | 19.7 (3.0) | - | 10 | - | MDD, GAD, other anxiety, depression, bipolar, NVLD, OCD | 0 | - | <$20,000/year 100% | Less than high school diploma 30%; High school diploma or GED 10%; Some college 30%; Bachelor degree 30% | No |
| Smith et al., 2019 | 224 | 21.86 (2.97)  [18-27] | Non-ID | 17.95 | - | Panic disorder, GAD, Separation disorder, Social Phobia disorder | 7.18 | 2.05 | - | Less than high school 26.42%; High school diploma/GED 41.06%; Some college 20.73%; Associate degree 4.47%; Bachelor degree 5.69% | No |
| Swain et al., 2015 | 69 | 20.5 (2.0) | Non-ID | 29 | - | - | 40.58 | 11.59 | - | - | No |
| Williams et al., 2021 | 947 | 30.94 (7.10) from SPARK participants and 24.09 (5.60) from laboratory community sample  [18-46] | Non-ID | 52.9% for SPARK sample; 39.4% for laboratory sample | - | Anxiety, depression, ADHD, PTSD, OCD | 21.3% in SPARK sample; 13.6% in laboratory sample | - | - | less than high school (.63%), high school diploma (25.13%), some college (26.29%), 2 year college (9.93%), 4 year college (23.13%), graduate/professional degree (12.25%) | No |
| Williams et al., 2022 | 39 | 21.4 (2.7), black participants, 19.5 (2.7), white participants  [16-26] | Non-ID; ID | 17.9 | - | SLD, emotional disturbance, ID, other health impairment, depression | 33.33 | - | - | - | No |
| Zamzow et al., 2016 | 20 | 21.39 (4.55)  [15-31] | Non-ID | 5 | none | - | - | - | - | - | No |
